# Mediastinal cystic teratoma misdiagnosed as pleural tuberculosis: A case report and review of 53 cases revealed by pleural effusion

**DOI:** 10.1002/ccr3.4139

**Published:** 2021-06-10

**Authors:** Patrick D. M. C. Katoto, Liliane N. Byamungu

**Affiliations:** ^1^ Division of Respiratory Medicine & Centre for Tropical Diseases and Global Health Department of Internal Medicine Catholic University of Bukavu Bukavu Congo; ^2^ Department of Medicine and Centre for Infectious Diseases Faculty of Medicine and Health Sciences Stellenbosch University Cape Town South Africa; ^3^ Department of Paediatric University of KwaZulu‐Natal Durban South Africa; ^4^ Department of Paediatric Catholic University of Bukavu Bukavu Congo

**Keywords:** dysembryoma, extra‐pulmonary tuberculosis, low‐income setting, thorax

## Abstract

Mediastinal teratoma (MT) can be misdiagnosed for a long period and revealed by fatal pleural effusion at any age. In high burden tuberculosis settings, it is important to consider MT for extra‐pulmonary tuberculosis not responding to medical treatment.

## Graphical abstract

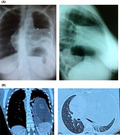

## 1 INTRODUCTION

Mediastinal cystic teratoma is a rare diagnosis in adolescence from a low‐income setting. Pleural effusion can often mislead infections and delay the resection. We report a case from a high burden tuberculosis country and analyze data from the literature. Clinical record of an adolescent patient from the Democratic Republic of Congo reported here and 53 records from other institutions identified via Medline from its inception to November 2017 were analyzed. We described demographic, clinic‐pathologic characteristics, and surgery outcomes and follow‐up of patients. Of 54 cases, 59% were females (mean (SD) age: 26 (12) years) and mostly from high‐income countries. Cough, shortness of breath, fever, and chest pain were mostly reported. Misdiagnosis was common, with periods between the onset of clinical manifestations to accurate diagnosis ranging from 1 week to 2 years. Our case was treated twice as tuberculosis. Duration of hospitalization varied from 4 days to 6 months and septic as well neurologic complications were frequently reported. With a follow‐up period ranged from 1 week to 2 years, five percent of patients died after surgery and 26 percent relapsed. The present report expands the spectrum of our knowledge showing the scarcity of reported mediastinal cystic teratoma in low‐income countries and that uncontrolled pleural effusion should help evoking the diagnosis.

A teratoma is defined as a type of germ cell tumor that may contain several different types of tissue such as hair, muscle, and bone.^1^ It may be immature or mature, based on how normal cells looked under a microscope; sometimes it is a mixture of both. They may occur in different parts depending on the sex (testicles, ovaries) or either in the chest, nervous system, or abdomen. They may be benign or malignant.^1^, ^2^

Mediastinal teratomas are the most common extra‐gonadal germ cell tumors. They account for approximately 15% of anterior mediastinal masses in adults and 25% in children and 50%‐70% of mediastinal tumors.^3^, ^4^ They may be symptomatic through different ways either by mass effect, endocrine function impaired or by rupture creating pleural effusion.^3^, ^4^, ^5^, ^6^, ^7^, ^8^ The pleural effusion can often lead to a misdiagnosis of respiratory infections especially in low‐income settings. We report on case of a cystic teratoma in a 15‐year‐old girl from a low‐income setting treated twice as tuberculosis. We also performed a review on cystic mediastinal teratoma with pleural effusion in the literature. In addition to our patient, we reviewed. Fifty‐three records from other institutions identified via Medline from inception to November 2017 that were well documented. Our review focused on analysis of clinical presentations, pathology findings, surgery procedures, and immediate and follow‐up outcomes.

## 2 METHODS

### 2.1 Patients

**FIGURE 1:**
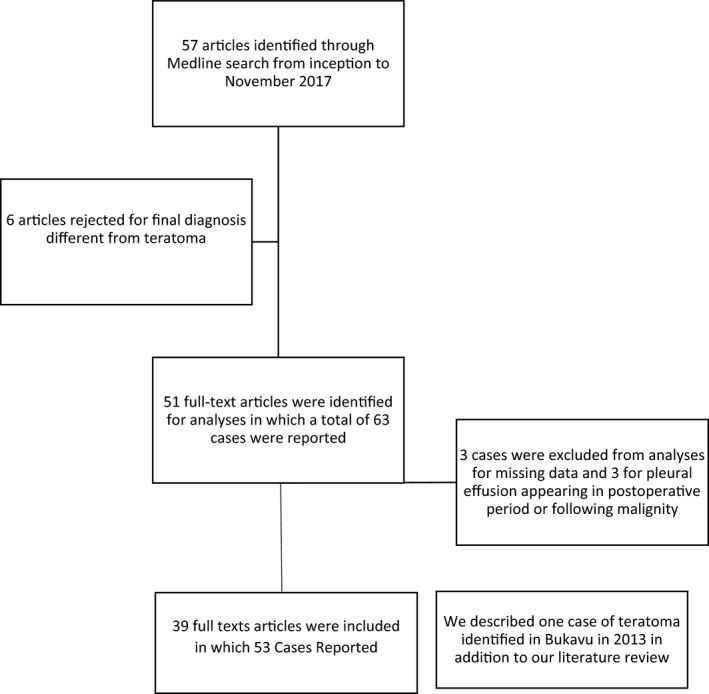
Flow Chart Diagram of literature search of cases presenting a mature teratoma with pleural effusion through Medline between May 2017 and November 2017

All cases were identified through Medline from different institutions using predefined MeSh terms [mediastinal teratoma] OR [mature mediastinal teratoma] OR [mature teratoma] OR [immature teratoma] AND [pleural effusion] from database inception to November 2017 (Figure 1). Mediastinal teratoma was diagnosed by anatomo‐pathologic findings after surgery resection or during medico‐legal expertise. The records of 54 patients were used for clinical analysis. We excluded 10 patients from whom clinical details and surgery outcomes were not obtained through our search. From full‐text articles, we extracted age of the patient at diagnosis, sex, country, first symptoms developed, type of surgery, anatomo‐pathologic findings (mature/immature, benign/malignant, type of tissue found) as reported and patients' outcomes after surgery (death/relapse). The time to diagnosis was calculated from the first onset of respiratory symptoms and the confirmation of diagnosis through histological analysis after surgery resection or autopsy. Chest X‐ray and CT examination were the major tools for establishing preoperative diagnosis. Descriptive analysis was essentially conducted for the report of the literature review. Categorical variables were expressed as proportions and continuous variables were expressed by means and standard deviations (±SD). Data were captured on a Microsoft Excel spreadsheet and exported to STATA version 14.1 (StataCorp LP, College Station, Texas 77 845 USA). Written consent was secured from our patient prior the study commenced.

## 3 RESULTS

### 3.1 Case report

#### 3.1.1 Patient presentation

**FIGURE 2:**
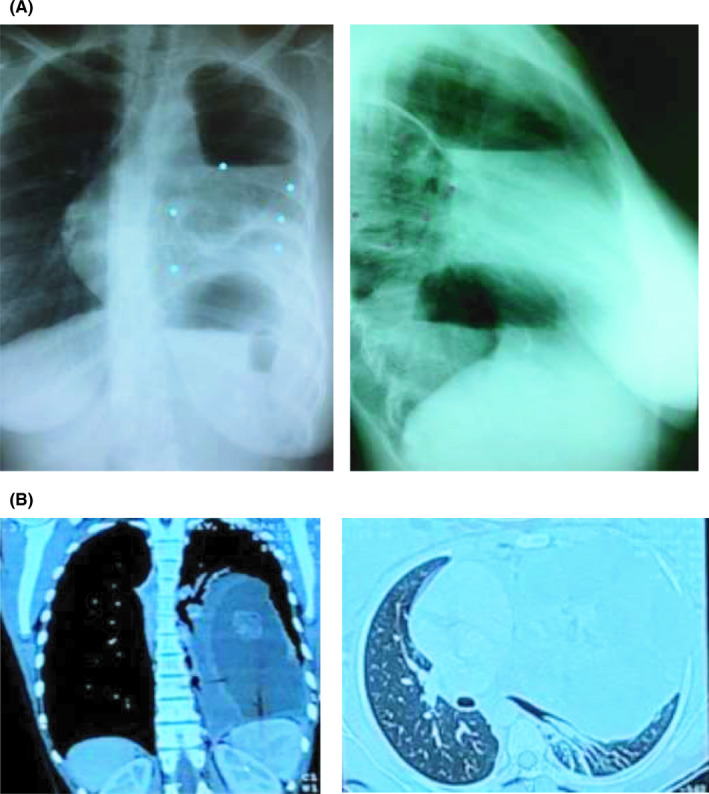
Chest X‐ray and CT Scan: a 15‐y‐old girl presenting with a large teratoma expressed by pleural effusion in South Kivu, Democratic Republic of Congo

A 15‐year‐old girl was transferred to our hospital with pleural effusion symptomatology along with respiratory distress (RD) which had occurred on multiple occasions. Her respiratory distress could have been relieved only by repetitive pleural tapping as much as 36 times for the last 3 years at the sequence of every 4 weeks before the actual transfer. She came from a rural setting in the East of DR Congo and resided in an informal settlement. Her medical history acknowledged that she was born healthy to nonconsanguineous parents, the sixth of eight healthy children. She apparently started developing the symptoms 3 years prior to the transfer with an increasing shortness of breath (SB) requiring medical assistance and incapacitated her to pursue studies and to accomplish ordinary activities. She had then been twice on 6 months of antituberculosis drugs first line regimen and had completed the treatment 6 months before the transfer. According to the medical report that accompanied the patient, she was treated for extra‐pulmonary tuberculosis with negative microscopy in accordance with national recommendations after both smear and pleural fluid were negative for Zielh‐Neelsen test and respiratory symptoms did not resolve after nonspecific antibiotic therapy. Of note, culture and GeneXpert testing could not have been conducted in the patient's geographical area nor samples been sent elsewhere due to political instability. On examination, weight was 53.2 kg, blood pressure: 100/70 mm Hg, heart rate: 82/min, and respiratory rate: 22/min and 36.5°C. Our clinical assessment confirmed a woody note and a silent auscultation on the left thorax. The right thorax presented a normal vesicular breath sound. Hemoglobin was slightly low: 11 g/dL (range: 12‐15) and both hepatitis C antibodies and hepatitis B surfaces antigen were nonreactive. The chest X‐ray (Figure 2A) and the CT‐Scan (Figure 2B) revealed a giant mediastinal mass with a collapsed left lung and consolidation. In brief, there was evidence of a large well defined complex cystic mass lesion involving antero‐superior mediastinum and extending to left to occupy the entire left hemithorax. The lesion consists of solid component with fatty elements, calcic foci, and multiple loculated cystic areas with intervening thick septae. In post–contrast scan, the solid component as well as the septae reveal definite enhancement. Few air loculi were seen within the mass lesion and many might have been related to previous diagnostic procedures. The lesion measured approximately 18.4 cm (SI) × 16.4 cm (AP) × 14.4 cm (Tr). There was partial atelectasis of the entire left lung, more so the lower lobe and seen compressed posteriorly. Mediastinum was shifted to the right side and lying lateral to the midline. However, mediastinal vessels were appeared normal as well as the right lung. No obvious focal lung lesion, septal thickening, or brochectatic changes were seen on the right side. A minimal left pleural reaction was noted.

#### 3.1.2 Surgical procedure

The surgery by left posterolateral thoracotomy noted a dense adhesion between the mass and the chest walls, pericardium and left lung. The left lower lung lobe was completely deflated and incarcerated by the mass with islands of epithelial tissue, hair, and calcified tissue. A total resection of the mass and decortication of the left lung were then conducted. Anatomopathological findings led to the diagnosis of a mediastinal mature cystic teratoma. The patient was then transferred to the intensive care unit for 3 days and required a prolonged stay in the wards due to the collapsed left lung. The latter required flexible bronchoscopy twice along with prolonged chest tube drained in situ. The chest tube was finally removed 15 days later after few trials of chest tube clamping followed by chest X‐ray.

#### 3.1.3 Patient evolution

No other complication occurred during the intervention. With adequate antibiotics prescribed for 14 days along with planed physiotherapy exercises and diet restrictions, the patient did not present any late complications (infection, respiratory distress) or story of collapsed lung. The patient was then discharged with antalgic that she took for almost 3 weeks. She stays in the city within a benevolent family to avoid exposition to biomass fuel smoking and to reduce risk of pulmonary infections inherent to the rural area. After 5 years of follow‐up, our patient did not complain about respiratory symptoms and she is now starting her first year at university.

### 3.2 Review of 63 cases of mature teratoma with pleural effusion

Fifty‐three cases were reported and analyzed from our search findings. 59% were females and mean (±SD) age was 26 (±12) years. Most of them come from high‐income countries. Cough, shortness of breath, fever, and chest pain were mostly reported. Most cases were misdiagnosed with time to first clinical symptoms ranged from 1 week to 2 years. Further, our case was first treated twice as tuberculosis (different regimens). About 91% showed an immature aspect while 16% were malignant. Ovarian and pancreatic tissues were mostly retrieved as content. Duration of hospitalization varied from 4 days to 6 months and septic as well neurologic complications were mostly reported. With a follow‐up period (of half of cases) ranged from 1 week to 2 years, roughly five percent of patients died after surgery and 26 percent relapsed (Table 1).

**TABLE 1:** Demographic, clinic‐pathologic, and survival findings of reported cases with mediastinal cystic teratoma with pleural effusion

Title	Age	Sex	Country	Clinics at presentation^a^	Pathology	Time to diagnosis	Type of surgery	Hospitalization	Recovery time	Evolution
Children under 1 y old										
Agarwal A et al, 2017 (Agarwal et al 2018)	28 wk GA	NS	NS	Large pericardial effusion	Encapsulated immature teratoma	4 wk	Pericardiocentesis and surgery resection (EXIT procedure)	NS	NS	NS
Dorterler ME et al, 2016 (Dorterler, Boleken, and Koçarslan 2016)	7 mo	F	NS	Persistent pulmonary infection and SOB exacerbated at rest	Benign mature teratoma	20 d	Right posterolateral between 4 and 5 interthoracal thoracotomy	NS	NS	Good after 3 mo FU
Gobbi D et al, 2007 (Gobbi et al 2007)	31 wk GA	F	Italy	Sudden symptomatology: cardiorespiratory distress, diffuse edema, abdominal distension, and no heart murmur	Encapsulated, multilobulated, cystic soft immature teratoma	26 d	Median sternotomy with resection of the mass	3 wk	5 mo	Good after 12 mo FU
Grebille AG et al, 2003 (Grebille et al 2003)	30 wk GA	F	France	Fetal hydrops	Pericardial teratoma	3 wk	Thoracoamniotic shunting	6 mo	15 d	Good after 6 mo FU
Children and adolescents between 1 and 17 y old										
Montebello A et al, 2017 (Montebello et al 2017)	17 y	M	NS	SOB and pleuritic pain	Benign cystic teratoma	2 mo	Thoracotomy and resection of the mass on the left after re‐accumulation of the fluid previously drained	NS	NS	Good with no recurrence
Tanupriya A et al, 2016 (Agrawal et al 2016)	12 y	M	NS	Intermittent shoulder and chest pain	Mature mediastinal cystic teratoma with subtotal unidirectional pancreatic differentiation	NS	Extensive resection	NS	NS	Good
Kuroda H et al, 2014 (Kuroda et al 2014)	16 y	F		Severe right chest pain and dyspnea	Mature cystic teratoma	2 y	Thoracoscopy resection	4 d		Good after 6 mo FU
Miyazawa M et al, 2012 (Miyazawa et al 2012)	15 y	M	Japan	Sudden onset of left‐sided severe chest pain and dyspnea	Cystic mature teratoma	5 mo	Thoracotomy with combined partial resection of the left brachiocephalic vein and mediastinal pleura	NS	NS	Good on FU
Sarkar A et al, 2010 (Sarkar et al 2010)	14 y	M	India	Heaviness of the left side of the chest side and SOB	Immature Teratoma	3 mo	Left thoracotomy was performed and a large anterior mediastinal tumor removed	NS	NS	Loss to FU
Kimura C et al, 2003 (Kimura et al 2003)	17 y	F	Japan	Anterior chest pain	Mediastinal mature teratoma perforating into the lung	NS	Total resection of the tumor with adherent parts of the left lung	NS	NS	Good on FU
Matsubara K et al, 2001 (Matsubara et al n.d.)	12 y	F	Japan	Severe chest pain and respiratory distress	Benign mediastinal cystic teratoma into the right pleural cavity	NS	Thoracotomy	NS	NS	Good on FU
Matsubara K et al, 2001 (Matsubara et al n.d.)	14 y	F	Japan	Severe chest pain and respiratory distress	Benign mediastinal cystic teratoma into the right pleural cavity	NS	Thoracotomy	NS	NS	Good on FU
Krishnan S et al, 1983 (Krishnan et al n.d.)	17 y	F	USA	Chest pain	Benign cystic teratoma with many Charcot‐Leyden crystals	NS	Surgery	NS	NS	NS
Adults above 17 y old										
Mohd Esa NY et al, 2016 (Mohd Esa et al 2016)	41 y	M	NS	Cough, weight loss, appetite, intermittent fever, intermittent pleural effusions	Benign cystic teratoma	1 y	Thoracotomy and resection	NS	Long	Unusual bacterial infection of the thoracotomy wound
Acharya MN et a, 2016 (Acharya et al 2016)	24 y	F	NS	Cough productive of green sputum, dyspnea, fever	Differentiated teratoma without malignant transformation (2.0 × 7.8 × 4.5‐cm)	2 mo	Posterolateral right thoracotomy extended through the same intercostal space	30 d	28 d	Good after 3 mo FU
Gautam M et al, 2016 (Mandal et al 2016)	28 y	F	India	Progressive SOB, heaviness of chest and low‐grade fever for last 1 mo	Mature cystic mediastinal teratoma with left sided pleural effusion	1 mo	Left anterolateral thoracotomy with debulking of mediastinal mass	37 d	30 d	Good on regular FU
Liu CH et al, 2014 (Liu et al 2014)	23 y	M	NS	History of fever, dyspnea, and right‐sided chest pain	Mature cystic mediastinal teratoma complicated by superior vena cava syndrome, acute mediastinitis, and pleural effusion	6 d	Urgent median sternotomy with resection	NS	NS	Good on FU
Chow MB et al, 2014 (Chow and Lim 2014)	24 y	F	China	Increasing SOB	Mature teratoma	4 mo	Resection by thoracotomy	NS	NS	Good on FU
Inoue Y et al, 2011 (Inoue et al 2011)	29 y	M	NS	Gradually worsening vague pain in the left chest	Mediastinal mature	7 d	Median sternotomy necessitating partial resection of the left upper lobe with a stapler	9 d	NS	Good after 2 y FU
Machuca JS et al, 2010 (Machuca et al 2010)	51 y	F	USA	Intermittent cough and precordial chest pain associated with SOB	Mature cystic teratoma	2 mo	Left thoracotomy	NS	NS	Good after FU
Yang CJ et al, 2007 (Yang et al 2007)	45 y	F	Taiwan	No symptoms	Ruptured cystic teratoma	2 y	Video‐assisted thoracoscopic surgery	8 d		Good after 10 mo FU
De Castro MA Jr et al, 2007 (de Castro et al n.d.)	27 y	F	Brazil	Chest pain and progressive dyspnea	Benign mediastinal teratoma	NS	Exploratory thoracotomy	NS	NS	Good after FU
Yuri T et al, 2006 (Yuri et al 2006)	19 y	M	Japan	Fever and dyspnea	Mixed choriocarcinoma and mature teratoma	7 d	Autopsy	0	NS	Died after acute respiratory failure
Yang WM et al, 2005 (Yang, Chen, and Lin n.d.)	45 y	F	Taiwan	Chest pain and dyspnea after accident	Ruptured cystic mature teratoma	11 d	Left lateral thoracotomy	1 d	10 d	Good after FU
Mori T et al, 2005 (Mori et al 2005)	20 y	F	Japan	Chest pain and palpitations	Mature teratoma	1 mo	Median sternotomy	5 d	5 d	Good after 23 mo FU
Kogure Y et al, 2005 (Kogure et al 2005)	28 y	M	Japan	Sudden onset of chest pain	Mature mediastinal cystic teratoma	3 wk	Thoracotomy with resection	NS	NS	Good on FU
Kogure Y et al, 2005 (Kogure et al 2005)	36 y	F	Japan	Sudden onset of chest pain	Mature teratoma	17 d	Thoracotomy with resection	NS	NS	Good on FU
Popp G et al, 2003 (Popp and Dragnev 2003)	27 y	M	USA	Progressive SOB	Mature teratoma, as well as pleural nodules with adenocarcinoma	NS	Thoracotomy and excision	NS	NS	Good on FU
Beduneau G et al, 2002 (Beduneau et al 2002)	25 y	M	France	Chest pain, fever	Benign mature mediastinal teratoma	Several months	Diagnostic mediastinotomy	NS	NS	Good on FU
Nagata K et al, 2002 (Nagata et al 2002)	27 y	M	Japan	Cough	Mature teratoma	NS	Thoracotomy	NS	NS	
Panicker JN et al, 2001 (Panicker et al 2001)	26 y	F	India	Catamenial dry cough and wheeze	Mediastinal teratomatous cyst with luteinized ovarian tissue	NS	Exploratory thoracotomy	NS	NS	Good on FU
Smahi M et al, 2000 (Smahi et al 2000)	12 patients with mean 32 y	7 F^5 M	France	Chest pain was present in 10 cases; cough, dyspnea, and septic episodes were present in 5 cases	Mature teratoma	NS	Posterolateral thoracotomy in 11 cases and an anterior thoracotomy in one case, pneumonectomy in 1 case, basal segmentectomy in 1 case and thymectomy in 1 case.	NS	NS	Morbidity included 2 phrenic nerve palsies, 1 pyothorax after pneumonectomy, 1 case of bleeding and 1 pleural effusion. No recurrences have been observed 5 to 87 mo FU
Yamamoto T et al, 1999 (Yamamoto et al n.d.)	27 y	M	Japan	NS	Immature teratoma	NS	Thoracotomy with resection	NS	NS	After 2 y of FU, transformation of teratoma into rhabdomyosarcoma in Klinefelter syndrome resulting in death
Ooshima M et al, 1999 (Ooshima et al 1999)	23 y	M	Japan	Right‐sided chest pain	Matured teratoma	NS	Thoracotomy with resection	NS	NS	Good on FU
Yamamoto N et al, 1996 (Yamamoto et al 1996)	43 y	F	Japan	Sudden chest pain	Mature cystic teratoma	NS	Thoracotomy with resection	NS	NS	
Minami H et al, 1994 (Minami et al 1994)	50 y	F	Japan	Cough and chest pain, serum CA19‐9 level was high (204.4 U/ml)	Mature teratoma	NS	Resection of the tumor with adherent part of the right lung	NS	NS	Serum CA19‐9 level returned to normal
Suster S et al, 1994 (Suster, Moran, and Koss 1994)	Mean 23 y	3 M and 1 F	USA	Cough, chest pain, dyspnea, and left‐sided pleural effusion	Two cases of solid variant of alveolar rhabdomyosarcoma, one case of embryonal rhabdomyosarcoma and one of pleomorphic rhabdomyosarcoma	NS		NS	NS	Recurrence and metastases within first 6 mo for all. Three died within this period, and one was lost to FU
Hiraiwa T et al, 1991 (Hiraiwa et al 1991)	27 y	F	Japan	Chest pain	Ruptured mediastinal cystic teratoma	NS	Surgical resection	NS	NS	Good on FU
Yeoman LJ et al, 1990 (Yeoman, Dalton, and Adam n.d.)	24 y	F	UK	Acute chest symptoms	Mediastinal teratoma with fat fluid	NS	Surgical resection	NS	NS	Good on FU

Abbreviations: EXIT, ex utero intrapartum therapy; FU, follow‐up; GA, gestational age; NS, not specified; SOB, shortness of breath.

^a^ Presence of pleural effusion was mandatory for inclusion even if note cited.

## 4 DISCUSSION

To our knowledge, there has no previous review of literature reporting on mediastinal cystic teratoma with pleural effusion. We found that mature teratoma with pleural effusion presented a high proportion in female patients during their young adult life. However, the literature search demonstrates the occurrence of mature teratoma with pleural effusion in patients as young as at 25 weeks of gestation and as old as 51 years.^2^, ^9^, ^10^, ^11^, ^12^, ^13^, ^14^, ^15^, ^16^, ^17^, ^18^, ^19^, ^20^, ^21^, ^22^, ^23^, ^24^, ^25^, ^26^, ^27^, ^28^, ^29^, ^30^, ^31^, ^32^, ^33^, ^34^, ^35^, ^36^, ^37^, ^38^, ^39^, ^40^, ^41^, ^42^, ^43^, ^44^, ^45^, ^46^, ^47^, ^48^, ^49^, ^50^, ^51^, ^52^, ^53^, ^54^, ^55^, ^56^, ^57^, ^58^, ^59^, ^60^, ^61^ Respiratory symptoms are predominant and not specific which has led often to a delay in the diagnosis for as long as 2 years.^2^, ^9^, ^10^, ^11^, ^19^, ^20^, ^22^, ^28^, ^30^, ^32^, ^33^, ^34^, ^35^, ^36^, ^37^, ^38^, ^39^, ^40^, ^41^, ^42^, ^43^, ^44^, ^45^, ^46^, ^47^, ^48^, ^49^, ^50^, ^51^, ^52^, ^53^, ^54^, ^55^, ^56^, ^57^, ^58^, ^59^, ^60^ The majority of studies were reported in high‐income countries. The requirement for the diagnosis to be made by CT scan and histopathology might explain the fact that such diagnosis is mostly reported in high‐income countries. Tissues found in the tumor appeared mostly immature and of ovarian and pancreatic origin. Except for cases that presented complications, the duration of hospitalization was short with variable lengths of follow‐up periods. Complications related to infections and metastases were reported in less than 10 cases. Relapses occurred for some cases and death was a rare outcome and was reported in 10 cases due to respiratory complications essentially.^17^, ^22^, ^24^, ^32^

The major strength of our study is the systematic search of literature and the number of cases reported. This will allow a more systematic assessment of the prevalence of the disease and could raise awareness among clinicians from low and middle‐income countries. Our results have highlighted a substantial proportion of cases from low‐income settings. Ascertainment misclassification following difficulties to diagnose mediastinal cystic teratoma might preside over the wrong impression of its rarity in poor settings. Regarding the high burden of respiratory infection diseases in such a region, clinicians might consider mediastinal tumors after an attempt to treat an infection disease not specified otherwise.

Although a systematic search of literature through Medline, among limitations of our study are the nonsystematic search of ≥3 databases and the exclusion of studies without full texts. Another limitation was related to the nature of the study that reported only on cases thus not representative of the populations from which the cases came from. However, the review has highlighted the need to consider such a differential diagnosis when assessing a patient with signs of pleural effusion especially in early childhood or young adult life.

## 5 CONCLUSION

Not all is about tuberculosis in sub‐Saharan Africa. We describe an original report of a giant mature cyst teratoma that required excision with left lung decortication in a young female Congolese after she has been treated twice as having pulmonary tuberculosis. This is the first documented case in the region. Diagnosis is difficult via noninvasive procedure, but clinicians should bear it in mind as a differential diagnosis to avoid delaying surgery.

## CONFLICT OF INTEREST

The authors declare that they have no competing interests.

## AUTHORS' CONTRIBUTION

LB: conceived and designed the study, supervised the patient follow‐up, searched for reported cases, drafted the initial manuscript, and revised the manuscript after feedback. PDMCK: contributed to design the study, supervise patient care, collect the data, conceptualize the report, and review and revise the manuscript. All authors read and approved the final manuscript.

## ETHICAL APPROVAL

Written informed consent was given by the patient and by her guardians for their clinical records to be used in this review.

## CONSENT FOR PUBLICATION

We received written informed consent from the patient and from her guardians to publish the information in this review.

## Data Availability

The datasets used and/or analyzed during the current study are available from the corresponding author on reasonable request.
